# Genome-wide RNAseq study of the molecular mechanisms underlying microglia activation in response to pathological tau perturbation in the rTg4510 tau transgenic animal model

**DOI:** 10.1186/s13024-018-0296-y

**Published:** 2018-12-17

**Authors:** Hong Wang, Yupeng Li, John W. Ryder, Justin T. Hole, Philip J. Ebert, David C. Airey, Hui-Rong Qian, Benjamin Logsdon, Alice Fisher, Zeshan Ahmed, Tracey K. Murray, Annalisa Cavallini, Suchira Bose, Brian J. Eastwood, David A. Collier, Jeffrey L. Dage, Bradley B. Miller, Kalpana M. Merchant, Michael J. O’Neill, Ronald B. Demattos

**Affiliations:** 1 Lilly Research Laboratories, Eli Lilly and Company, Indianapolis, IN USA; 2 Eli Lilly and Company Limited, Lilly Research Centre, Erl Wood Manor, Windlesham, Surrey GU20 6PH UK; 3 Sage Bionetworks, Seattle, WA USA; 4 Present address: Trans Thera Consulting Co, Indianapolis, IN USA; 5 Present address: AbbVie Deutschland GmbH & Co. K.G, Ludwigshafen, Germany

**Keywords:** Microglia, rTg4510, Tauopathy, RNAseq, Neuroinflammation, Alzheimer’s disease

## Abstract

**Background:**

Activation of microglia, the resident immune cells of the central nervous system, is a prominent pathological hallmark of Alzheimer’s disease (AD). However, the gene expression changes underlying microglia activation in response to tau pathology remain elusive. Furthermore, it is not clear how murine gene expression changes relate to human gene expression networks.

**Methods:**

Microglia cells were isolated from rTg4510 tau transgenic mice and gene expression was profiled using RNA sequencing. Four age groups of mice (2-, 4-, 6-, and 8-months) were analyzed to capture longitudinal gene expression changes that correspond to varying levels of pathology, from minimal tau accumulation to massive neuronal loss. Statistical and system biology approaches were used to analyze the genes and pathways that underlie microglia activation. Differentially expressed genes were compared to human brain co-expression networks.

**Results:**

Statistical analysis of RNAseq data indicated that more than 4000 genes were differentially expressed in rTg4510 microglia compared to wild type microglia, with the majority of gene expression changes occurring between 2- and 4-months of age. These genes belong to four major clusters based on their temporal expression pattern. Genes involved in innate immunity were continuously up-regulated, whereas genes involved in the glutamatergic synapse were down-regulated. Up-regulated innate inflammatory pathways included NF-κB signaling, cytokine-cytokine receptor interaction, lysosome, oxidative phosphorylation, and phagosome. NF-κB and cytokine signaling were among the earliest pathways activated, likely driven by the *RELA*, *STAT1* and *STAT6* transcription factors. The expression of many AD associated genes such as *APOE* and *TREM2* was also altered in rTg4510 microglia cells. Differentially expressed genes in rTg4510 microglia were enriched in human neurodegenerative disease associated pathways, including Alzheimer’s, Parkinson’s, and Huntington’s diseases, and highly overlapped with the microglia and endothelial modules of human brain transcriptional co-expression networks.

**Conclusion:**

This study revealed temporal transcriptome alterations in microglia cells in response to pathological tau perturbation and provides insight into the molecular changes underlying microglia activation during tau mediated neurodegeneration.

**Electronic supplementary material:**

The online version of this article (10.1186/s13024-018-0296-y) contains supplementary material, which is available to authorized users.

## Background

Microglia are tissue macrophages of the central nervous system (CNS) [1]. They help shape the neuronal circuits during CNS development and constantly survey the CNS environment in adulthood [2–5]. In response to neuronal damage or pathogenic stimuli, microglia become activated to serve as first line defenders. Proliferation, migration, and a range of morphological and functional transformations are the hallmarks of microglia activation [6–8]. In neurodegenerative diseases, such as Alzheimer’s disease (AD), activated microglia are detected by histological analyses of postmortem human brains [9, 10] and positron emission tomography (PET) imaging using TSPO (Translocator protein) ligands in living patients [11–13].

Genetic studies also suggest that microglia are directly involved in the disease cascade wherein they contribute to AD onset and development. Several single nucleotide polymorphisms (SNPs) associated with microglia and immune function genes, such as *TREM2, CD33, CR1, ABCA7, SHP1*, and *APOE*, significantly affect AD risk [14, 15]. Rare coding mutations in *PLCG2, ABI3,* and *TREM2* are also associated with increased risk for AD and other neurodegenerative diseases [16–18].

The exact biological roles of microglia in AD are not fully understood. It is generally thought that microglia activation can be both positive and deleterious [19, 20], wherein early in disease, microglia activation is considered beneficial due to increased motility and phagocytic activity that facilitates the clearance of pathological protein aggregates and promotes tissue recovery [21]. However, in later phases of neurodegeneration, chronic microglia activation with excessive and persistent pro-inflammatory cytokine release and oxidative species production is thought to be detrimental to neuronal function and survival [22–24]. These two opposite microglia phenotypes were traditionally categorized as classic (M1/pro-inflammatory) or alternative (M2/tissue repair) activation phenotypes, a concept derived from peripheral macrophage biology yet currently is under reconsideration [25]. Nevertheless, it is well known that microglia play a complex role in AD and that the longitudinal characterization of microglia molecular changes during disease progression is incredibly important.

Microglia activation has mainly been studied by examining morphological changes and measuring limited activation markers. Recently, genome-wide gene expression profiling has been used to characterize the molecular changes of isolated microglia from animal models of neurodegenerative diseases, including the amyloidosis models such as APPswe/PS1dE9 [26], PS2APP [27], and 5xFAD [28], and the amyotrophic lateral sclerosis (ALS) model SOD1^G93A^ (super-oxide dismutase) [29, 30]. These studies revealed that complex and dynamic molecular changes underlie microglia activation in response to pathological insults [31, 32].

However, microglia specific molecular changes in response to pathological tau perturbation have remained elusive. Filamentous tau accumulation is not only a pathological hallmark of AD, but also the characteristic of other tauopathies, such as progressive supranuclear palsy, frontotemporal dementias (FTD) and corticobasal degeneration [33]. It was reported that microglia activation preceded notable pathological tau accumulation in transgenic tau (P301S) models [34] and drove tau pathology [35]. A recent study using a microglia ablation animal model demonstrated that microglia mediate pathological tau propagation [36]. Therefore, it is important to understand how microglia respond to pathological tau perturbation at the molecular level.

In this study, we performed transcriptome profiling of acutely isolated microglia from a widely used animal model of tauopathy, rTg4510. In this model, human 4-repeat tau containing an FTLD-17 associated mutation (P301L) is expressed postnatally in forebrain neurons, which results in age-dependent pathological tau accumulation, neurodegeneration, and cognitive deficits [37, 38]. Microglia cells were acutely isolated from 2-, 4-, 6-, and 8-month old rTg4510 and wild type control animals to capture longitudinal transcriptome changes. Gene expression was profiled by RNA sequencing (RNAseq) and analyzed by statistical and systems biology approaches. Key genes and pathways were identified that underlie microglia activation in response to tau perturbation. In addition, differentially expressed microglia genes were compared to human brain gene expression networks.

## Methods

### Animals

All animals were housed under standard conditions with access to water and food ad libitum. All animal procedures and experiments were performed in accordance with the Institutional Animal Care and Use Guidelines for Eli Lilly and Company. C57/Bl6 mice were used for method development.

rTg4510 transgenic mice were generated as described by Ramsden et al. [38]. Female rTg4510 mice were licensed from the Mayo Clinic (Jacksonville Florida, USA) and bred for Eli Lilly by Taconic (Germantown, USA). In this mouse line, the human tau (P301L) gene is placed downstream of a tetracycline operon–responsive element (TRE). In the presence of a second transgene encoding a CaMKIIα-controlled tetracycline-controlled transactivator (tTA), tau is expressed in forebrain neurons postnatally but repressible by administration of the tetracycline analog doxycycline (dox). In this study, female mice containing both transgenes were used as tau transgenic (rTg4510), while wild type (WT) littermates that do not contain any transgene were used as control animals.

### AlphaScreen assays

AlphaScreen assays (Perkin Elmer Life Sciences) were developed as previously described [39] and performed according to the manufacturer’s guidelines using tau specific antibodies. Antibodies against total tau DA9 (amino acids 102–140), TG5 (amino acids 220–240), and conformationally changed tau, MC1, were kind gifts from Peter Davies (Albert Einstein College of Medicine, New York).

Brain cortex tissue samples collected from rTg4510 and wild type mice were lysed (in the absence of sarkosyl) and fractionated into soluble and insoluble fractions by low speed and high speed spin (100,000 g), using a protocol adapted from Berger et al. [40]. The insoluble fraction P1 (pellet after 100,000 g centrifugation) was subjected to AlphaScreen assays to quantify the levels of total tau and conformationally changed tau.

### Immunohistochemistry (IHC) and neuropathological characterization

rTg4510 and age-matched WT controls were anesthetized at specific time points and transcardially perfused with ice-old phosphate-buffered saline (PBS). The brain was removed and the right hemisphere was drop fixed in 10% buffered formalin and embedded in paraffin wax. Sagittal brain sections (6 μm) were deparaffinized and processed for IHC. Tissue sections were processed in an autostainer (720, Thermo Scientific) with the following steps: (1) 10 min 0.3% H_2_O_2_; 30 min normal goat serum (Vector Labs); (2) 60 min in primary antibody (PG-5, courtesy of Peter Davies; Iba-1,WAKO); (3) 30 min in biotinylated secondary antibody (goat anti-rabbit or goat anti-mouse, Vector Labs); (4) 30 min avidin-biotin complex solution (Vector Labs); (5) 5 min in 3,3′-diaminobenzidine (Vector Labs). Sections were counterstained with haemotoxylin prior to dehydration and cover-slipping. The stained slides were scanned and digitized using the Scanscope AT slide scanner (Aperio) at 20x magnification and viewed using Imagescope software (version 12.2.1.5005; Aperio). An automated algorithm was used to count the number of microglial cells in the region of interest. The number of PG-5 positive neurons was quantified manually using the digitized images.

### Evaluation of microglia isolation methods

Two microglia isolation methods were evaluated, a traditional Percoll gradient method [41, 42] and a newly-developed method of antibody mediated affinity magnetic cell separation. Mice were anesthetized and transcardially perfused with ice-cold PBS. Forebrains were dissected and kept in Hank’s Balanced Salt Solution (HBSS -Ca/-Mg, Thermo Fisher Scientific). Brain tissue was mechanically and enzymatically dissociated into a single cell suspension using a Neural Tissue Dissociation Kit on a gentleMACS® Dissociator following manufacturer’s protocol (Miltenyi Biotec, Bergisch Gladbach, Germany). Cells were then divided into two aliquots (Additional file 1: Figure S1A), one aliquot was subjected to Percoll gradient separation (GE healthcare, USA), and the other was subjected to myelin removal using 30% Percoll (GE healthcare, USA), followed by CD11b antibody-coupled MicroBeads and MACS® technique (Magnetic-activated cell sorting) according to the manufacturer’s protocol (Miltenyi Biotec, Bergisch Gladbach, Germany). The microglia cell layer from the Percoll gradient and CD11b-positive and -negative cells were collected for further evaluation by quantitative real-time reverse-transcription polymerase chain reaction (q-RT-PCR) or Fluorescence-activated cell-sorting (FACS) analysis. FACS analysis was done using PE-CD11b and FITC-CD45 antibodies (BioLegend). Briefly, cells were washed and incubated with antibodies for 30 min at 4 °C and then fixed. The next day, stained cells were analyzed using FACS/CALIBUR (BD Bioscience).

### RNA isolation

Microglia cell pellets were processed for total RNA isolation using RNeasy mini kits according to the manufacturer’s protocol (Qiagen). RNA samples were quantified using a Nanodrop (Thermo Fisher Scientific).

### Quantitative real-time reverse-transcription polymerase chain reaction (q-RT-PCR)

RNA samples were reversely transcribed into complementary DNA (cDNA) using TaqMan Reverse Transcription Reagents (Thermo Fisher Scientific, Waltham, MA, USA). cDNAs were subjected to q-RT-PCR analysis using Taqman assays (Thermo Fisher Scientific, Waltham, MA, USA).

### RNA sequencing, data quality control (QC), and gene mapping

Microglia were isolated using CD11b MicroBeads methods as described above. Total RNA were isolated and sent to Cofactor Genomics (St. Louis, USA) for RNA deep sequencing. RNA samples were first examined using Agilent Bioanalyzer (Agilent Technologies, Palo Alto, CA, USA) for purity and quality. RNAs were reverse transcribed to cDNA using Ovation RNA-Seq System Version 2 (NuGEN, San Carlos, CA) according to the manufacturer’s protocol. The resulting cDNAs were then sheared using a focused ultrasonicator (Covaris Inc., Woburn, MA, USA) and the libraries were prepared using the Kapa LTP Library Preparation Kit (Illumina, San Diego, CA, USA). RNAseq of 32 samples was performed on Illumina HiSeq2000 systems according to the manufacturer’s protocol. For each sample, approximately 50 million clusters (100 million reads) were generated via paired-end 100-bp reads.

RNAseq data were subjected to a QC pipeline developed at Eli Lilly and Company. Briefly, base quality/base composition, heterologous organism contamination, adapter content, mapping rate/mapped read counts, 3′ bias, template length, and rRNA/mitochondrial content were checked. Four samples, WT-4 m-3, rTg4510-4 m-3, WT-8 m-4, and rTg4510-8 m-4, were excluded from further analysis due to failed RNAseq QC assessment.

To map reads to genes and obtain gene level expression measures, RNAseq data were subjected to a “rollup” pipeline developed at Eli Lilly and Company. The following rules were applied for the rollup: (1) Exon reads of multiple assays from the same libraries were summed; (2) Exons were excluded if more than 80% of samples have less than 10 counts; (3) Robust gene level signals across exons of a gene were determined by a robust linear model and were output for each library and each gene; (4) Mean signal of log2 transformed gene level across all samples were median normalized.

Accession number for RNA-Seq data in Gene Expression Omnibus (GEO) is GSE123467.

### Statistical analysis of differentially expressed genes

Twenty-eight samples that passed QC were used for statistical analysis. Statistical analysis included a 2 × 4 genotype x month factorial linear model, followed by contrasts. Differentially expressed genes (DEGs) were defined using a cutoff of 1.5-fold of change and less than 5% false discovery rate (FDR). FDR was per contrast.

### Microglia transcriptome data sets in AD animal models

The Microglia transcriptome data from APPswe/PS1dE9 mouse model [26] and the DEG results were downloaded from Glia Open Access Database (GOAD) [43]. The microglia microarray data from 5xFAD mouse model [28] was download from NCBI GEO (GSE65067) and then re-analyzed to generate the DEG list. The criteria for DEG are the same across studies, i.e. adjusted *p*-value < 0.05 and |fold change| > 1.5.

### Principal component analysis (PCA) and hierarchy clustering analysis

Normalized and log2-transformed gene expression data from each sample was used. PCA and hierarchical clustering analysis were carried out using statistical software JMP with “Ward” method and “standardize data” options. Two-way clustering option was turned on after initial clustering analysis of samples to visualize gene expression patterns across samples.

### Pathway enrichment and gene set analysis

KEGG pathway enrichment analysis was performed for each group of genes using R package clusterProfiler [44, 45]. The p-value and Q-value cutoffs were 0.05 and 0.1 respectively.

Subsets of genes that have been associated with AD were selected based on previous literature reports or public data sources: AD risk genes by genetic study [14], phagocytosis, complement system, Scavenger receptors (SR) subsets (KEGG gene GO), and microglia classic or alternative activation (M1 or M2 states) signature genes identified from primary human microglia cells [46].

### Identification of upstream regulators

The upstream regulators that affected these DEGs were predicted using IPA’s “Upstream Regulator Analysis” tool [47]. Z-scores and *p*-values were used to select upstream regulators in the regulator network containing direct interactions between genes that exist in bone marrow cells and immune cells. Z-scores assess the match of observed and predicted up/down regulation patterns. *P*-values measure enrichment of the regulated genes in the dataset without taking into account the regulation direction in order to avoid incomplete and biased regulatory information used in z-score calculation. The top 10 common genes gated by *p*-values and z-scores were selected.

### Human transcriptomic network

A statistical network of gene co-expression using an ensemble network inference algorithm was constructed. Briefly, nine distinct gene co-expression network inference methodologies were applied, including ARACNe [48], Genie3 [49], Tigress [50], Sparrow [51], Lasso [52], Ridge [52], mrnet [53], c3net [54], and WGCNA [55]. The edge lists from each method were ranked based on the edge weights and a mean rank for each edge across methods was identified, then the total number of edges supported by the data with Bayesian Information Criterion for local neighborhood selection with linear regression was identified. The ensemble approach is inspired by work from the DREAM consortia [56] showing that ensemble methods are better at generating robust gene expression networks across heterogeneous data-sets. This method was applied to RNAseq data from 632 participants in the ROS/MAP (The Religious Orders Study/The Memory and Aging Project) [57, 58], to identify a gene co-expression network associated with aging and late onset Alzheimer’s disease.

### Network cell type specificity annotation

The cell type specific RNAseq data sets derived by Zhang et al. [59] were used to annotate the networks in terms of cell specific markers in Fig. 7a. Multiple distinct clusters in the human co-expression network were identified to be associated with cell types including microglia (blue), endothelial cells (red), astrocytes (cyan), neurons (yellow) and myelinating oligodendrocyte cells (magenta). Strong associations between cell type specific markers and co-expression signatures in post-mortem brain tissue were discovered.

### Network module identification

Network modules were identified based on the inferred network topology with a consensus clustering algorithm [60] applied to multiple individual module identification methods. Individual network clustering methods applied to the network topology include CFinder [61], GANXiS [62], a fast greedy algorithm [63], InfoMap [64], LinkCommunities [65], Louvain (http://iopscience.iop.org/article/10.1088/1742-5468/2008/10/P10008/meta), Spinglass [66], and Walktrap (https://arxiv.org/abs/physics/0512106) methods. After the consensus modules were identified, gene set enrichment analyses were performed on consensus modules, as discussed below.

### Comparison of the rTg4510 microglia DEGs with human network modules

Differential expression genes at each time point in the rTg4510 microglia were run via ortholog conversions and compared with the human microglia module 5 and module 9 using enrichment analysis and visualized using Cytoscape (http://www.cytoscape.org). The strength and significance of overlap is represented by Q-values and odds ratios (OR).

## Results

### Evaluation and validation of mouse brain microglia isolation methods

To select a microglia isolation method for transcriptome profiling, two microglia isolation methods were evaluated, a traditional Percoll gradient method, and a newly-developed antibody mediated affinity magnetic cell separation method (Additional file 1: Figure S1A, Methods). Using q-RT-PCR, microglia-specific markers, such as AIF1, CX3CR1, and CD11b, were shown to be mostly enriched in CD11b-positive cells (Additional file 1: Figure S1B, upper panel), while the non-microglia markers, such as NeuN, GFAP, and SOX10, were barely present (Additional file 1: Figure S1B, lower panel). To examine whether these isolation procedures cause artificial stimulation of microglia, the expression levels of genes that associate with microglia activation, including TNFα and IL-1β, were analyzed using q-RT-PCR and normalized to AIF1. Compared to total isolated cells, CD11b positive cells have comparable gene expression levels for TNFα and IL-1β, suggesting CD11b bead isolation procedure caused minimal stimulation of microglia (Additional file 1: Figure S1C). Further evaluation using Fluorescence-activated cell-sorting (FACS) indicated that more than 90% of magnetic bead isolated cells were CD11b positive and CD45 low (Additional file 1: Figure S1D), which is consistent with the published microglia surface marker characteristics [41]. Based on these results, the CD11b antibody coupled magnetic beads separation method was used to acutely isolate microglia from mouse brain in the following experiments.

### Microglia isolation from rTg4510 mice

**Fig. 1:**
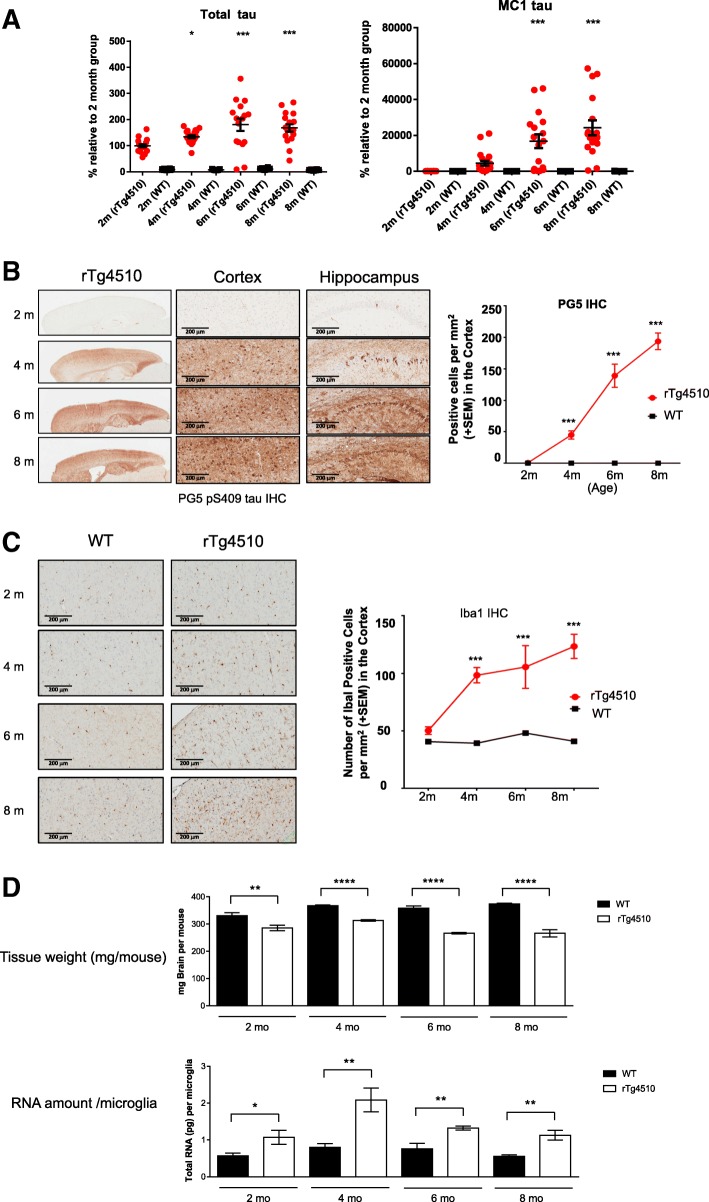
Pathological study and microglia isolation from rTg4510 mice. **a**. AlphaScreen assays showing levels of insoluble total tau and MC1-reactive tau in the cortex of rTg4510 (red circles) and wild-type (WT, black circles) mice over time (2-, 4-, 6- and 8- months); expressed as a percent relative to the 2-month old rTg4510 mice. Data are mean ± SEM (20 mice per group); statistical analysis: one-way ANOVA analysis + Dunnet’s test, *,**,*** = *p* < 0.05, 0.01, 0.001 vs. 2-month old rTg4510 group. **b**. Immunohistochemistry (IHC) using PG5 antibody to detect phospho-S409 tau accumulation in 2-, 4-, 6-, and 8- month old rTg4510 mouse brain. Quantification result using number of positive cells in the region of interest is plotted on the right (*** *p* < 0.005 two way factorial ANOVA). **c**. Iba1 IHC of the cortex region of rTg4510 and age-matched WT mice. The number of Iba1 positive cells are quantified and plotted on the right (*** *p* < 0.005 two way factorial ANOVA). **d**. Microglia isolation summary. Bar graphs show the weight of forebrain tissue (upper panel), and the amount of total RNA per microglia cell (lower panel) of rTg4510 and WT animals at different age. Data are mean +/− SEM; statistical analysis: Student *t* Test, *, **, **** = *p* < 0.05, 0.01, 0.0001 WT versus rTg4510 at each age

In the rTg4510 animal model, human 4-repeat tau (P301L) is expressed postnatally in the forebrain neurons. Progressive age-related accumulation of pathological tau tangles, neuronal loss, and behavioral impairments were reported previously [37, 38]. To understand the time course of tau pathology development in rTg4510 in our hands, the insoluble P1 fraction of the cortices from rTg4510 and wild type (WT) mice over time (2-, 4-, 6- and 8- months) were analysed using AlphaScreen assays (Methods). As expected, elevated levels of total tau (DA9) were detected in rTg4510 compared to WT (Fig. 1a, left panel). The levels of conformationally changed tau (MC1) in rTg4510 were first detected above WT levels at 4 month of age and continued to accumulate at 6 and 8 months of age (Fig. 1a, right panel).

To further evaluate the tau pathology at cellular level, immunohistochemistry (IHC) was performed using PG5 (phospho-tau S409) antibody (Methods). At 2 months of age, a very limited PG5 positive neurons were detected in the cortex and the hippocampal region of rTg4510 (Fig. 1b), but by 4 months of age, substantial level of pathological tau was observed and continued to increase at 6 and 8 months (Fig. 1b). Similar results were observed with other tau antibodies including MC1, AT-8, PHF-1, and nY29 (data not shown).

Along with these pathological changes, the number of microglial cells, indicated by positive Iba1 staining, increased dramatically in the forebrain of rTg4510 in comparison to WT controls starting from 4 months of age (Fig. 1c).

In order to compare microglia transcriptome change across tau pathological continuum, four age groups of rTg4510 and WT animals, 2-, 4-, 6-and 8-month old, were selected for microglia isolation to capture longitudinal changes. Four biological replicates were prepared for each genotype at each time point. Acute microglia isolation was performed using pooled forebrain tissues dissected from 8 to 10 animals. As summarized in Fig. 1d, forebrain tissue weight was significantly decreased in rTg4510 compared to WT, consistent with tissue atrophy (Fig. 1d, upper panel). Interestingly, the level of total RNA per microglia cell was higher in rTg4510 than that in WT, at as early as 2 months of age, suggesting increased transcriptional and/or translational activity in microglia at this early stage (Fig. 1d, lower panel).

### Genome-wide RNAseq of acutely isolated rTg4510 microglia

**Fig. 2:**
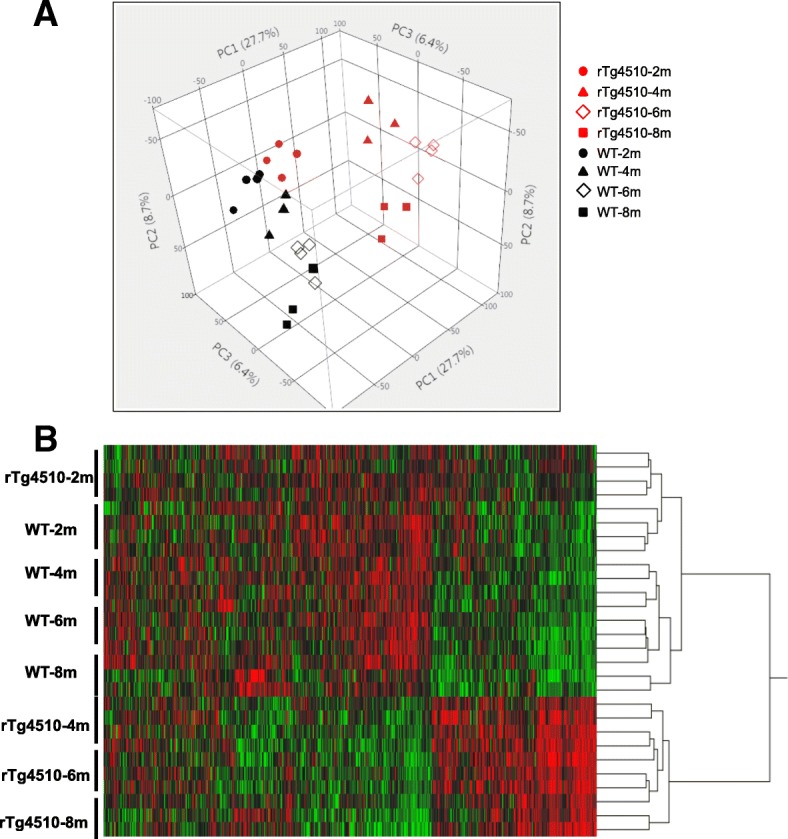
Genome-wide transcriptome analysis of acutely isolated rTg4510 microglia. **a**. Principal component analysis (PCA) of all 18,588 transcripts from 28 samples. Plot shows three-dimensional comparison of transcripts in four age groups and two genotypes of microglia cells. Data were transformed by logarithm of base 2. **b**. Heat map display of the clustering analysis result of all 18,588 transcripts. All data on logarithm of base 2 from 28 samples and hierarchical analysis was carried out in statistical software JMP

Forebrain microglia RNA samples were subjected to deep RNA sequencing (Methods). Approximately 100 million reads for each sample were collected and 18,588 genes were mapped. Principal component analysis (PCA), using all mapped genes, showed significant separation between rTg4510 and WT groups, which emerged at 2 months of age and became more evident in later age groups. The variance within biological replicates was small (Fig. 2a). The first component of PCA is highly associated with genotype and the second component is associated with the age of the groups. The first PCA component accounts for 27.7% of the total variance, suggesting that gene expression signature in rTg4510 microglia is significantly different from that of WT microglia.

Hierarchical clustering analysis divided all samples into two major clusters. The first cluster consists of the 2-month rTg4510 samples and all WT samples, and the other cluster consists of the remaining rTg4510 samples, indicating that the significant separation of rTg4510 microglia transcriptome from WT microglia started at 4 months of age (Fig. 2b).

### Identification of differentially expressed genes (DEGs)

**Fig. 3:**
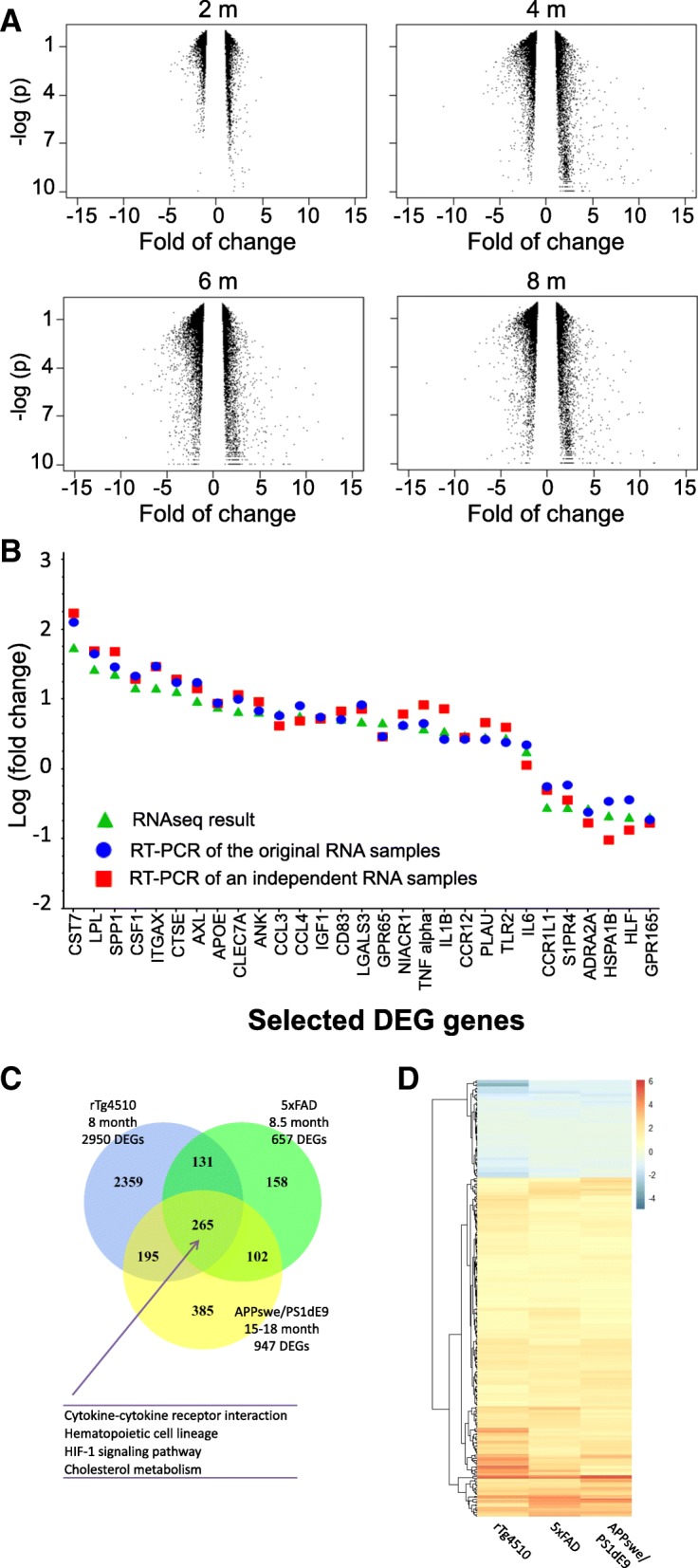
Identification and validation of differential expression genes (DEGs). **a**. Volcano plot of DEGs in rTg4510 transgenic microglia relative to WT microglia at indicated age. Fold change are plotted against the –log(*p* value). The vertical empty space indicates the 1.5 fold change cutoff threshold. **b**. Validation of selected DEGs by q-RT-PCR. Twenty eight DEGs, 22 up-regulated and 6 down-regulated ones, were selected for q-RT-PCR. Log (fold change, rTg4510 vs. WT microglia RNA) of q-RT-PCR results of the original RNA samples (circles) and an independent set of RNA samples (squares) are plotted against RNAseq results (triangulars). Genes are ordered from left to right based on highest to lowest fold change values of RNAseq results. **c**. Venn diagram of the number of DEGs in the three studies as labelled. The number of common DEGs is shown in the overlapping areas. Enriched KEGG pathways are listed on bottom. **d**. Heat map of genes common to the three studies. The color intensity represents the log2 fold change of the expression

A total of 4672 genes were differentially expressed (DEG) in rTg4510 microglia versus WT across all age groups (FDR < 0.05 and |fold change| > 1.5, Table 1 and Additional file 2: Table S1). There were more up-regulated genes than down-regulated genes at each time point (Table 1 and Fig. 3a). At 2 months of age, only 368 genes were differentially expressed in rTg4510 microglia. A majority of them display fairly small magnitude of changes with absolute values of fold change less than 2 (Fig. 3a and Table 1). However, more than two thousand genes were DEGs at 4 months of age and maintained at 6 and 8 months of age. Consistent with the PCA and clustering analysis, DEGs at 4-, 6-, and 8- months of age were not significantly different from each other, indicating that the most gene expression change occurred between 2 to 4 months of age.

**Table 1:** Distribution of 4672 DEGs (FC > 1.5) in rTg4510 microglia

Month	DEG	Up	Down	|Fold change| > 2	|Fold change| < = 2
Month 2	368	293	75	70	298
Month 4	2564	1760	804	1286	1278
Month 6	3689	2101	1588	2036	1653
Month 8	2950	1952	998	1665	1285

To confirm the DEGs identified by RNAseq, the top 22 up-regulated genes and 6 down-regulated genes were selected for q-RT-PCR confirmation. Microglia RNAs purified from an independent cohort of 4-month old animals, together with original RNA samples, were used for q-RT-PCR. The fold changes (FC) of these 28 genes detected by q-RT-PCR were highly consistent with the RNA sequencing results (Fig. 3b).

To understand how microglia transcriptome change in response to pathological tau in comparison to the change in response to amyloid pathology, we downloaded or generated DEG lists from two previously published microglia transcriptome studies using mouse models with β-amyloid deposition, the APPswe/PS1dE9 model [26] and the 5xFAD model [28], and compared them to the DEGs from the 8-month old rTg4510 (Additional file 3: Table S2). Across the three studies, 265 genes were consistently differentially expressed in response to tau or amyloid pathology (Fig. 3c and Additional file 3: Table S2). Furthermore, all 265 DEGs showed the same direction of change although with different magnitude of change, with 206 up-regulated and 59 down-regulated genes (Fig. 3d). Pathway enrichment analysis of the 265 common DEGs found that four KEGG pathways were significantly enriched, cytokine-cytokine receptor interaction (Q-value =0.0088), hematopoietic cell lineage (Q-value =0.0094), HIF-1 signaling pathway (Q-value =0.00016), and cholesterol metabolism (Q-value =0.00016), suggesting that these common genes and pathways in microglia are involved in response to both β-amyloid and pathological tau (Fig. 3c).

### Pathway enrichment analysis of DEGs

**Fig. 4:**
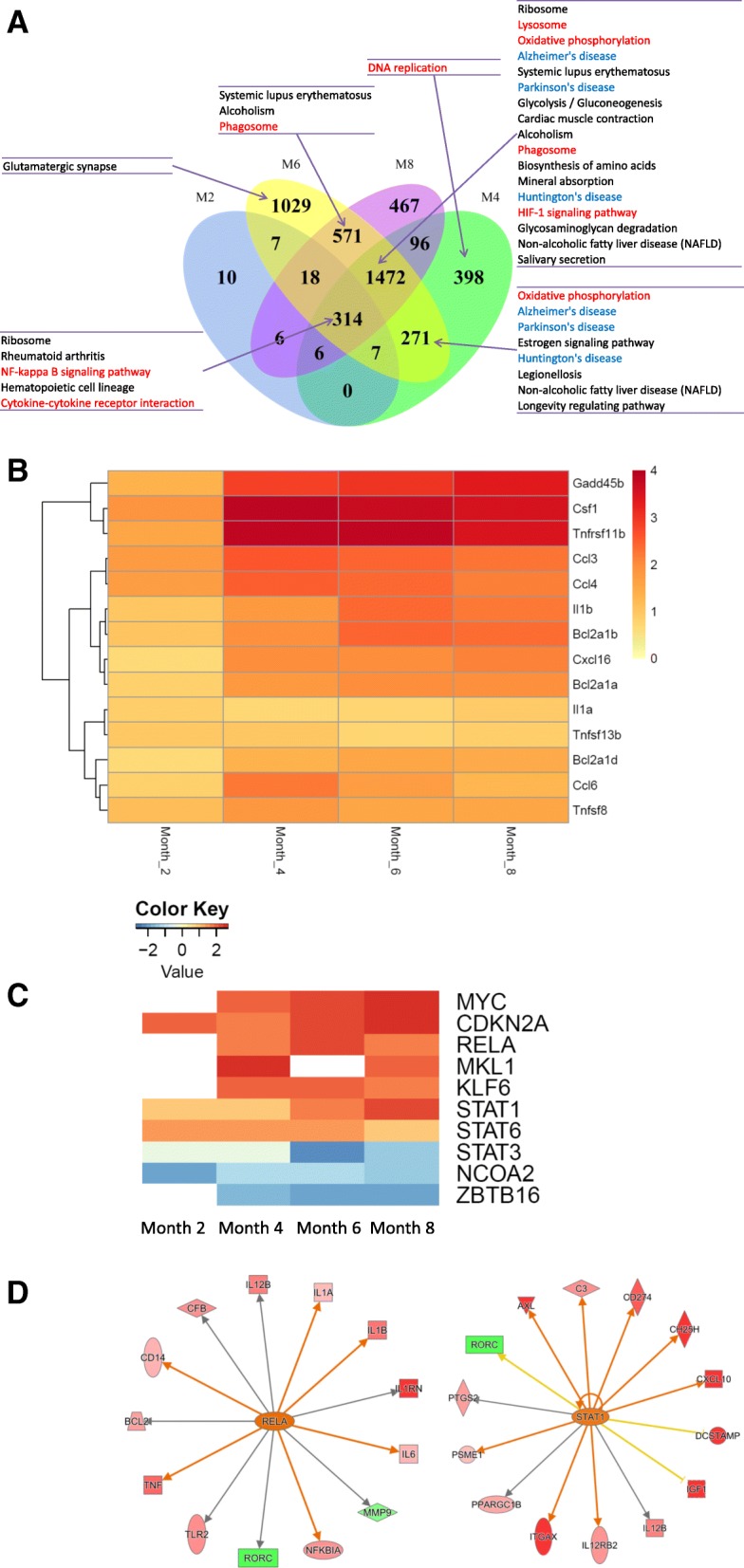
Bioinformatics analysis of DEGs. **a**. Venn diagram of the number of DEGs in each age group. The number of common DEGs across months is shown in the overlapping areas. Subgroups of genes shown in the diagram are subjected to KEGG pathway enrichment analysis. Enriched KEGG pathways are listed on the side and connected to the region by arrows. **b**. Heat map of genes involved in NF-κB signaling and cytokine-cytokine receptor interaction. The color intensity represents the log2 fold change of the expression. **c**. Upstream regulator analysis. Z-score heat map of upstream regulators. The color intensity represents the Z-score. **d**. IPA network of target genes regulated by RELA (left panel) or STAT1 (right panel). Red represents up-regulation and green represents down-regulation. The color intensity represents the level of change

DEGs at 2 months of age represented the early-responders to tau pathology in microglia. Out of the 368 DEGs at 2 months, 314 genes (85.33%) remained differentially expressed at 4-, 6-, and 8- months of age (Venn diagram, Fig. 4a), and majority of them (261 genes) were continuously up-regulated (Additional file 4: Figure S2 heat map of the 314 genes). KEGG pathway analysis showed that the innate inflammatory pathways, e.g., NF-κB signaling and cytokine-cytokine receptor interaction, are enriched in these 314 genes, suggesting that these two pathways were among the first to be activated and remained active over the course of tau pathology development. Genes involved in these two pathways include several tumor necrosis factor superfamily (*TNFSF*) genes, *TNFRSF8, TNFRSF11B* and *TNFSF13B*, two interleukin 1 family (*IL-1*) genes, *Il1a* and *Il1b*, four chemokine genes, *CCL3, CCL4, CCL6* and *CXCL16*, three B cell leukemia/lymphoma 2 related (*BCL2*) genes, *BCL2A1A, BCL2A1B* and *BCL2A1D*, and *CSF1* and *GADD45B*. The expression changes of these genes are shown in Fig. 4b.

At 4 months of age, additional inflammation-related pathways became activated, including oxidative phosphorylation, lysosome, HIF-1 signaling, and phagosome pathways (Table 2, KEGG at each month). In addition, the neurodegeneration disease related pathways, AD, PD (Parkinson’s disease) and HD (Huntington disease), were enriched in the 1742 DEGs common to 4-, 6-, and 8- months of age, suggesting similar immune/microglia mechanisms underlying these neurodegenerative conditions (Fig. 4a and Table 2). The 398 DEGs specific to 4 months of age were enriched in the DNA replication pathway, aligning well with the microglia number increase observed at this age (Fig. 4a and Fig. 1c).

**Table 2:** Number of DEGs involved in selected KEGG pathways at each age group. Values in the parentheses are Q-values of pathway enrichment test, and significant Q-values (< 0.1) are marked with^a^

Pathways	Month 2	Month 4	Month 6	Month 8
NF-κB signaling	7 (9.69E-03^a^)	21 (8.74E-03^a^)	18 (2.44E-01)	24 (2.83E-03^a^)
cytokine-cytokine receptor interaction	11 (1.86E-02^a^)	42 (1.82E-02^a^)	53 (1.07E-02^a^)	49 (4.68E-03^a^)
Lysosome	4 (4.73E-01)	37 (3.96E-08^a^)	40 (1.13E-06^a^)	41 (8.58E-09^a^)
Alzheimer’s disease	3 (7.53E-01)	45 (1.41E-08^a^)	56 (2.20E-10^a^)	39 (1.51E-04^a^)
Parkinson’s disease	0	30 (2.75E-04^a^)	42 (3.43E-07^a^)	26 (2.51E-02^a^)
Huntington’s disease	0	33 (8.74E-03^a^)	47 (8.59E-05^a^)	27 (3.54E-01)
Oxidative phosphorylation	0	37 (1.41E-08^a^)	47 (4.71E-11^a^)	33 (3.82E-05^a^)
HIF-1 signaling pathway	3 (6.34E-01)	25 (1.12E-03^a^)	25 (2.51E-02^a^)	23 (2.26E-02^a^)
Phagosome	3 (7.91E-01)	31 (1.89E-03^a^)	45 (2.40E-04^a^)	43 (5.11E-05^a^)

Genes involved in NF-κB signaling (Additional file 5: Figure S3A) and AD pathways (Additional file 5: Figure S3B) are illustrated in more detail using the Pathview package [67]. Each gene rectangle is split into four bins to represent the 4 age groups. In the NF-κB signaling pathway, the expression of 26 out of 104 genes was significantly altered in rTg4510 microglia, including pro-inflammatory cytokines, *IL1b* and *TNFalpha*. In addition, the expression of *IκBalpha* was up-regulated implicating a negative feedback response (Additional file 5: Figure S3A). In the AD related pathway, 62 out of 177 genes showed differential expression including *APOE* and *LPL*. The expression of *BACE1* and *PSEN1,* which encode two enzymes critical for the production of pathogenic Aβ, was also affected (Additional file 5: Figure S3B).

### Identification of upstream regulators

To identify the upstream regulators that drive the transcriptome changes and pathway activation in rTg4510 microglia, 4672 DEGs were analyzed using IPA’s “Upstream Regulator Analysis” tool [47]. The top 10 upstream regulators are shown in Fig. 4c. *RELA, STAT1, STAT3,* and *STAT6* are key mediators of the immune responses, while *MYC, CDKN2A, MKL1, KLF6* and *ZBTB16* regulate the cell proliferation which represents another aspect of microglia activation. These upstream regulators control multiple downstream targets and mediate broader gene expression changes (Fig. 4d).

### Clusters of DEGs in rTg4510 microglia

**Fig. 5:**
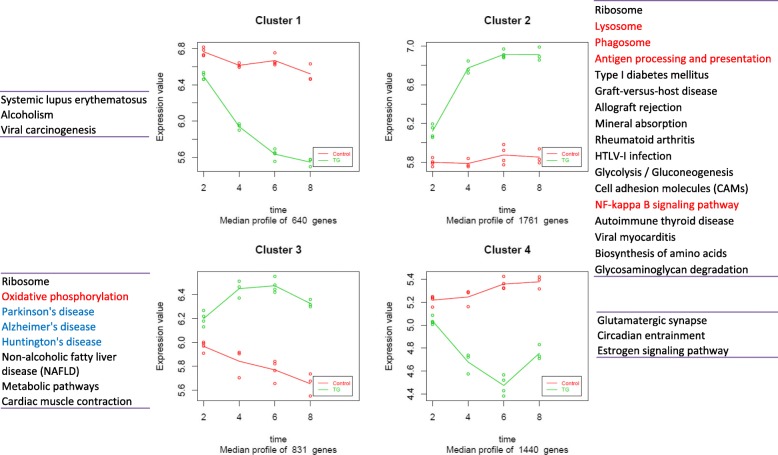
Clusters of the DEGs in rTg4510 microglia. The total 4672 DEGs in rTg4510 were classified into four major clusters using hierarchical clustering based on the correlations of expression profiles. In each plot, X axis represents the age (month) and y axis represents the normalized gene expression value (log2 converted). Each plot represents the overall expression profile of the genes in one cluster. The dots are the median expression values of genes in each replicates within the cluster, while the line indicates the median expression values of genes in the cluster. Lines representing WT are in red, whereas lines for transgenic are in green. The enriched KEGG pathways are listed besides each cluster

Based on their longitudinal expression changes in rTg4510 microglia, the 4672 DEG were divided into four major clusters using Pearson’s correlation coefficients between pairs of genes (Fig. 5). Cluster 1 includes 640 genes (13.7% of total DEGs) whose expression was down-regulated in rTg4510 microglia with age, but remain fairly stable in WT microglia. The second cluster, the largest cluster, includes 1761 genes (37.7% of total DEGs). Their expression was continuously up-regulated in rTg4510 microglia across four age groups but remained stable in WT microglia. Enrichment analysis revealed that genes in this cluster are mostly related to innate inflammatory pathways and microglia functions, such as lysosome, phagosome, antigen processing and presentation, and NF-κB signaling pathways. The third cluster includes 831 genes (17.8% of total DEGs), which were up-regulated in rTg4510 with peak expression at 4 or 6 months of age, but their expression was down-regulated in WT microglia. Several neurodegenerative disease-related pathways are enriched in this cluster. The last cluster includes 1440 genes (30.8%) whose expression is significantly down-regulated in rTg4510, but moderately up-regulated in WT microglia. Notably, genes involved in glutamatergic synapse belong to this cluster.

### Expression pattern of selected genes

**Fig. 6:**
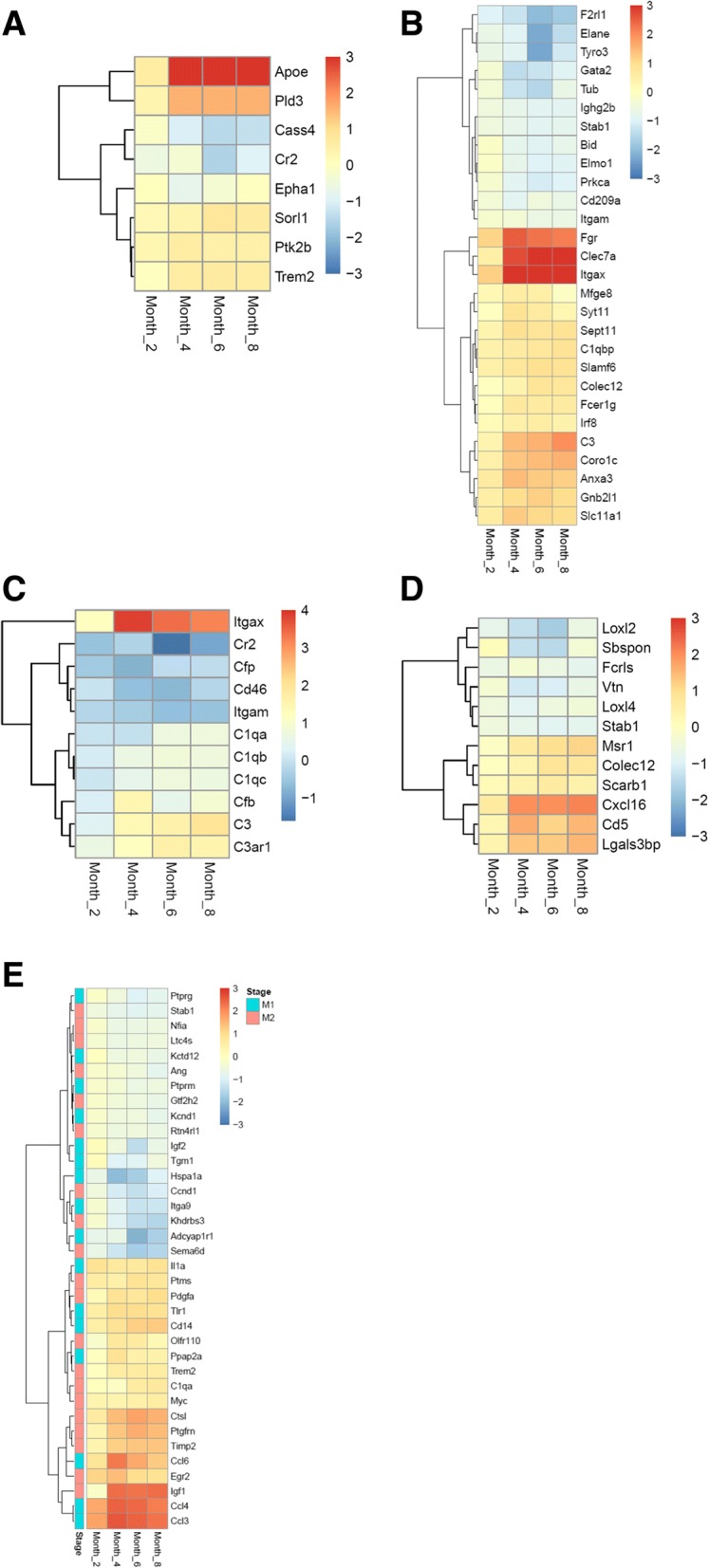
Expression analysis of selected gene sets. Heat maps of (**a**). AD risk genes, (**b**). Phagocytosis genes, (**c**). Complement components, (**d**). scavenger receptors, and (**e**). M1 and M2 specific genes. Only differentially expressed genes are shown in the heat maps. The color intensity represents the log2 fold change of the expression in rTg4510 versus WT microglia. M1 or M2 specific genes were noted blue or red on the far left column, respectively

Genes that are associated with AD genetically or biochemically were further analyzed and their expression changes are shown as heat map in Fig. 6. Out of 26 AD risk genes [14, 15], eight were differentially expressed in at least one age group (Fig. 6a). *APOE, PLD3, PTK2B, SORL1* and *TREM2* were up-regulated, while *CASS4, CR2* and *EPHA1* were down-regulated. *APOE* has the highest fold change among them (about 8 fold at 4 months of age).

Phagocytosis is one of the primary functions of microglia and is implicated in amyloid plaque clearance [68]. Twenty-eight of the phagocytosis genes (*N* = 113, see Methods) showed differentially expression (Fig. 6b). *FGR, CLEC7A* and *ITGAX* showed dramatic up-regulation in rTg4510 at 4-, 6- and 8- months (fold change > 5).

Complement components were shown to be up-regulated surrounding amyloid plaques in human AD [69, 70], and mediate early synapse loss in AD animal models [71]. In rTg4510 microglia, genes encoding the three subunits of C1q, namely *C1qa, C1qb,* and *C1qc*, complement factor (*Cfb*) in the alternative complement pathway, and downstream components *C3* and *C3AR1*, were significantly up-regulated (Fig. 6c).

Scavenger receptors (SR) participate in cellular adhesion and immune response, and microglia scavenger receptors are associated with the development of AD [72–74]. Twelve out of 28 SR genes were differentially expressed in at least one age group. Half of the SR DEGs were constantly up-regulated, while the other half were down-regulated in all age groups (Fig. 6d). Gene Ontology (GO) analysis showed that all the up-regulated SRs are located on the plasma membrane (GO:0005886), whereas a majority (except *FCRLS*) of the down-regulated SRs are secreted proteins (GO:0005615, extracellular space).

Although the M1/M2 paradigm of microglial activation is being reconsidered, we explore whether there is apparent M1/M2 polarization in rTg4510 microglia and whether there is M2 to M1 transition in the course of pathology advancement. The expression of M1 and M2 signature genes [46] were examined in rTg4510 microglia transcriptome. Sixteen out of the 38 (42%) M1 specific genes, including *TNFαlpha* and *IL1b*, and 20 out of the 40 M2 (50%) specific genes, including Arginase 1 and YM1, were differentially expressed in at least one of the age groups, mixed with up-regulation and down-regulation (Fig. 6e). Within each age group, the ratios of affected M1 genes and affected M2 genes are similar (Fisher’s exact test, *P*-value = 0.37), suggesting no clear polarization to either M1 or M2 states at any of the 4 ages. Additionally, no difference in the expression pattern of M1 and M2 DEGs was observed across different age groups (Chi-squared test, P-value = 0.838), arguing against the hypothesis that there is M2 to M1 phenotype transition at least within the studied time period. Taken together, rTg4510 microglia became activated with a distinct gene expression signature from M1 or M2 states.

### rTg4510 microglial DEG sets overlap with human co-expression modules

**Fig. 7:**
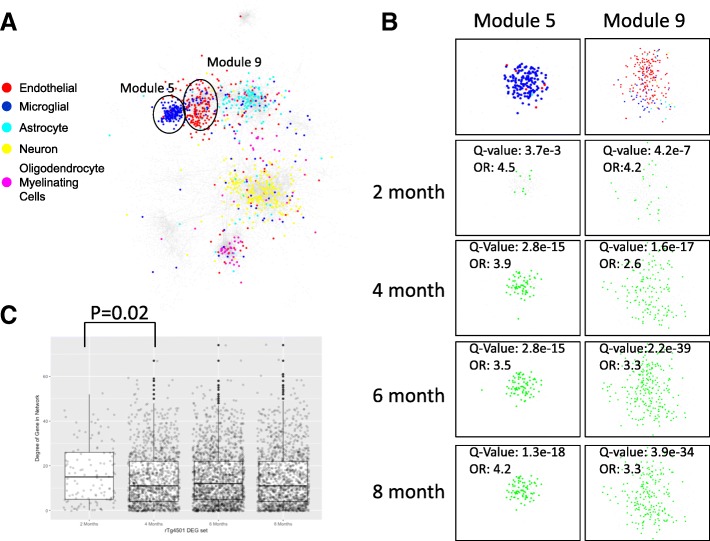
Comparison of rTg4510 microglia DEGs with human gene expression network. **a**. Human brain gene co-expression network was constructed using RNAseq data from 632 participants in the ROS/MAP (Methods). Cell type specificity of genes was annotated: microglia (blue), endothelial cells (red), astrocytes (cyan), neurons (yellow) and myelinating oligodendrocyte cells (magenta). Network module 5 enriched of microglial signature genes and module 9 enriched of endothelial genes are labelled. **b**. DEGs in rTg4510 microglia (green) at 2-, 4-, 6-, and 8-months significantly overlap with human network module 5 and 9 (based on Fisher’s exact test). The significance and strength for the overlap is shown as Q-value and odds ratio (OR). **c**. Connectivity of rTg4510 DEGs at 2-, 4-, 6-, and 8- months in human co-expression network. The mean connectivity of rTg4510 microglia DEGs is represented by degree of genes shown in box plot. DEGs at two months are more connected (i.e. ‘hub’ like) than those at later time points (*P*-value 0.02, Wilcoxen rank sum test)

In order to understand how relevant the gene expression changes in rTg4510 microglia are for human biology, we performed network analysis using RNAseq data from human brains, and mapped the DEGs in the rTg4510 microglia onto the human networks. RNAseq data from the dorsolateral prefrontal cortex of 632 participants in ROS/MAP cohorts were used to develop a human transcriptional co-expression network using an ensemble approach (Methods). Using cell type specific gene expression data published by Zhang et al. [59], the genes in the network were annotated by cell type and indicated by different colors, including neuronal, astrocytic, endothelial, microglia, and oligodendrocyte cell types (Fig. 7a). Transcriptome modules were identified based on the inferred network topology and multiple distinct modules in the network associated with cell types (Methods).

Enrichment analysis of rTg4510 microglia DEGs against this human co-expression network revealed that rTg4510 DEGs overlapped with human microglia co-expression module (module 5) and endothelial module (module 9) (Fisher’s exact tests). The enrichment became increasingly significant at later ages, represented by lower Q-value (Fig. 7b). However, the strength of overlap in terms of the odds ratio (OR) from a Fisher’s exact test is strongest at two months (OR: 4.5 and 4.2 for human Modules 5 and 9 respectively) (Fig. 7b).

In gene expression networks, highly connected genes are more likely to represent genes that are critical to regulatory processes in the cell or upstream drivers of response to disease [51, 75]. We hypothesized that early response genes in rTg4510 microglial at 2 months of age are more connected in the human network. To explore this, the degree of genes (the number of connected genes) was calculated and compared across different time points. Indeed, the mean connectivity of DEGs at 2-months is higher than those at later time points (*P*-value 0.02, Wilcoxen rank sum test), see Fig. 7c, suggesting 2-months DEGs are more critical and more ‘hub’ like in the network.

## Discussion

In this study, we performed longitudinal genome-wide gene expression profiling of rTg4510 microglia cells and identified 4672 DEGs. System biology approaches revealed that NF-κB signaling and cytokine-cytokine receptor interaction pathways were the first to be activated, likely driven by the key upstream regulators *RELA*, *STAT1* and *STAT6*. DEGs belong to four clusters based on their longitudinal expression changes. The major cluster of DEGs contain innate inflammatory genes that were continuously upregulated. We also developed human transcriptomic co-expression networks and demonstrated that rTg4510 mouse microglia DEGs overlapped with the human network sub-modules.

To our knowledge, this is the first study to assess longitudinal gene expression changes in isolated microglia from tau transgenic animals. When compared to previous studies using isolated microglia from animal models of β-amyloid deposition, several similarities and differences were noted. Common genes and pathways were identified in spite of the different pathology in these models, intracellular neuronal tau accumulation versus extracellular β-amyloid plaque deposition, suggesting that these genes and pathways present central and core mediators of microglia activation. The DEGs specific to each study may function as upstream sensors of different stimuli and/or fine-tune microglia activation toward the specific pathological condition in each animal model. The number of DEGs in the rTg4510 model is much larger than that in the amyloidosis models (2950 genes vs. less than 1000 genes). This could be due to technology differences since RNAseq used in this study is much more sensitive in detecting low-abundant RNAs than microarray method used in the amyloidosis studies [76]; however this may also reflect a different level of microglia activation in response to tau versus β-amyloid deposition. This RNAseq dataset generated in pure tau animal models not only brings complementary information to AD but also sheds light on the understanding of microglia activation in other tauopathies.

Using isolated microglia in transcriptome studies is advantageous to tissue level transcriptome investigations, because the latter is confounded by altered cellular composition, as illustrated by a recent study [27]. In rTg4510 mice, microglia number significantly increased compared to WT animals (Fig. 1 b and c), therefore using isolated microglia would identify microglia-specific gene expression changes other than expression changes caused by cell number alteration. A gene expression study of rTg4510 brain tissue by laser microdissecting specific regions of the hippocampus was published previously [77]. A preliminary comparison to that dataset indicated that the number of DEGs and the degree of change are significantly different, and DEGs only partially overlapped (unpublished results).

By analyzing the longitudinal changes of the 4672 DEGs and their biological functions, we started to understand the dynamic molecular changes that underlie microglia activation in response to tau pathology. Microglia are very sensitive to pathological disturbance. In 2-month old rTg4510 mouse brain, total tau was elevated with a very limited amount of pathological tau as assessed by biochemical and IHC methods. However, even at this early stage microglia activation was evident as shown by gene expression change and cell number increase. Inflammatory pathways including NF-κB signaling and cytokine-cytokine receptor interaction pathways are the earliest to be activated, likely driven by upstream transcription factors RELA in the NF-κB pathway and STATs in cytokine signaling pathway. Consistent with microglia number increase, a set of 4-month specific DEGs are enriched in DNA replication, indicating active microglia proliferation at this stage. The additional 1742 DEGs emerged at 4 months and remaining as DEGs at 6 and 8 months are enriched in oxidative phosphorylation, lysosome, HIF-1 signaling, and phagosome pathways (Fig. 4a and Table 2), as well as the neurodegeneration disease related pathways. This set of genes likely function as mediators to enhance and expand the microglia response. NF-κB is a well-known master regulator of inflammation [78]. Activation of NF-κB was found in several disease conditions, such as in AD [79]. Currently, multiple drug discovery activities targeting NF-κB and STAT family proteins are underway, including treating AD by inhibiting phosphorylation of STAT3 [80].

We also explored the expression changes of genes that have been linked to AD previously to help understand their functions in AD pathogenesis. We found that several of them, such as genetic risk factors *APOE, PLD3, TREM2*, phagocytotic genes *FGR, CLEC7A* and *ITGAX*, complement components and scavenger receptors (SR) are upregulated in rTg4510 microglia, suggesting activation of these genes during microglia activation. The expression of *APOE*, the first and the strongest genetic risk factor for late-onset AD, showed the biggest up-regulation among all AD risk genes with a ~ 8-fold increase in rTg4510 microglia cells at 4 months of age. Another AD risk gene *TREM2* is also upregulated. Recently, *TREM2-APOE* pathway was identified as a major regulator of microglia activation in response to amyloid pathology [81]. Lacking either *TREM2* or *APOE* resulted in reduced microglia response to plaque, altered plaque morphology, and increased neuronal dystrophy [28, 82, 83]. Both APOE and TREM2 are associated with lipid metabolism, thus their activation may connect with altered lipidomic homeostasis caused by AD pathology [28]. Components of the complement system were also up-regulated in response to tau pathology, including all three C1q components in the classical complement pathway, the complement factor (*Cfb*) in the alternative complement pathway, and downstream C3 and C3AR1 receptor. It was shown that C1q and C3 tagging of the damaged synapses were required for their elimination by microglia in diseased conditions [2]. In amyloidosis AD models, C1q levels were increased and the synaptic localization of C1q was detected even before plaques formation [71]. Up-regulation of complement components in rTg4510 might be a response to damaged synapses/neurons that need to be tagged for microglia elimination. Among up-regulated SRs, *MSR1 (SCARA1)* and *SCARB1 (SR-BI)* have been reported to mediate the clearance of β-amyloid fibrils [84–86]. Data here suggests that these two receptors may also be involved in the microglial response to tau and/or tau mediated neurodegeneration.

One limitation of this study is that only female animals were used for microglia isolation and transcriptome analysis, due to the size of this study and the availability of animals. It has been recently demonstrated that microglia gene expression and functional levels can differ between female and male mice. Female microglia express less pro-inflammatory genes and are neuroprotective in ischemia animal model [87]. In addition, in spared nerve injury (SNI) induced neuropathic pain model, microglia are required for pain hypersensitivity in male mice but not in female mice. Inhibiting microglia activity reversed the mechanical allodynia only in male mice [88]. These observations emphasized the importance of including both genders of animals in microglia studies. Therefore, future studies would be needed to evaluate tau-induced transcriptome change and molecular mechanism of microglia activation in male versus female animals.

Another potential limitation of our study is that we used pooled microglia for RNAseq analysis. It is possible that differential and distinct activation status existed at individual cell level. Following recent technical advances, a study using transcriptional single-cell sorting identified a novel microglia type associated with neurodegenerative diseases (DAM) in an amyloidosis animal model [89]. Similar studies on tau animal models are needed to help understand microglia activation at single-cell resolution, as well as the heterogeneity of microglia in the brain. In addition, multiple CNS cell types communicate and mutually depend on each other to function. The activity of microglia is especially linked to astrocyte function, and it was recently shown that microglia activation induces neurotoxic reactive astrocyte formation [90]. Therefore, comprehensive study of the molecular changes in different cell types, together with bioinformatics tools, are needed to further our understanding on neurodegenerative diseases and provide opportunities for novel therapeutic targets and biomarker identification.

## Conclusion

In response to pathological tau accumulation, microglia respond early and continuously by producing over 4000 gene expression changes. These gene changes drive the proliferation of microglia cells and the activation of key innate immune pathway, such as NF-κB signaling, cytokine-cytokine receptor interaction, lysosome, oxidative phosphorylation, and phagosome pathways. These gene expression changes highly overlapped with human co-expression modules, suggesting conserved gene expression regulation between animal models and human diseases. This study revealed temporal transcriptome alterations in microglia cells in response to pathological tau perturbation and provides insights to the molecular changes underlying microglia activation during tau mediated neurodegeneration.

## Additional files

Additional file 1: **Figure S1.** Microglia isolation from adult mouse brain and validation by q-RT-PCR and FACS analysis. (**A**). Graphic overview of two microglia isolation methods. (**B**). q-RT-PCT result of relative expression levels of microglia-specific markers (Iba1, CX3CR1 and CD11b) and non-microglia markers (GFAP, NeuN, and Sox10) in mouse brain tissue and different cell populations. (**C**). q-RT-PCT result of the expression levels of pro-inflammatory genes, TNFα and IL-1β, in CD11b positive cells versus in total cells and in microglia cells isolated by Percoll gradient method. (**D**). FACS analysis of total, CD11b-positive and CD11b-negative cells using PE-CD11b and FITC-CD45 antibodies. Left, Histograms of relative cell count for PE-CD11b antibody. (DOCX 153 kb)

Additional file 2: **Table S1.** The list of DEGs (FDR < 0.05 and |fold change| > 1.5) at each age groups of rTg4510. (XLSX 970 kb)

Additional file 3: **Table S2.** The list of DEGs in three microglia transcriptomic studies. (XLSX 240 kb)

Additional file 4: **Figure S2.** Heat map of the 314 genes that were differentially expressed at all ages. The color intensity represents the log2 fold change. (DOCX 72 kb)

Additional file 5: **Figure S3.** (A). NF-kappa B signaling pathway and (B). AD pathway overlaid with gene expression change in rTG4510 versus WT microglia**.** The pathview plots show the expression change in log (fold change, rTg4510 vs. WT) of genes in the pathway. Each gene rectangular is split into four bins with filled colors, representing the log2 fold change at the four time points. (DOCX 200 kb)

## Abbreviations

AD: Alzheimer’s disease
ALS: Amyotrophic lateral sclerosis
CNS: Central nervous system
DEG: Differentially expressed gene
FACS: Fluorescence-activated cell-sorting
FC: fold changes
FDR: False discovery rate
FTD: Frontotemporal dementia
HD: Huntington disease
IHC: Immunocytochemistry
OR: odds ratio
PCA: Principal component analysis
PD: Parkinson’s disease
PET: Positron emission tomography
RNAseq: RNA sequencing
ROS/MAP: The Religious Orders Study/The Memory and Aging Project
SNPs: Single nucleotide polymorphisms
SOD: Super-oxide dismutase
SR: Scavenger receptors
TSPO: Translocator protein
