# Supplementary material for: Genome-wide RNAseq study of the molecular mechanisms underlying microglia activation in response to pathological tau perturbation in the rTg4510 tau transgenic animal model
Source: Mol Neurodegener. 2018 Dec 17;13:65. doi: 10.1186/s13024-018-0296-y (PMC6296031; doi:10.1186/s13024-018-0296-y)
Supplement: Supplementary file 3 [file 13024_2018_296_MOESM5_ESM.docx]

Wang H. et al

A.

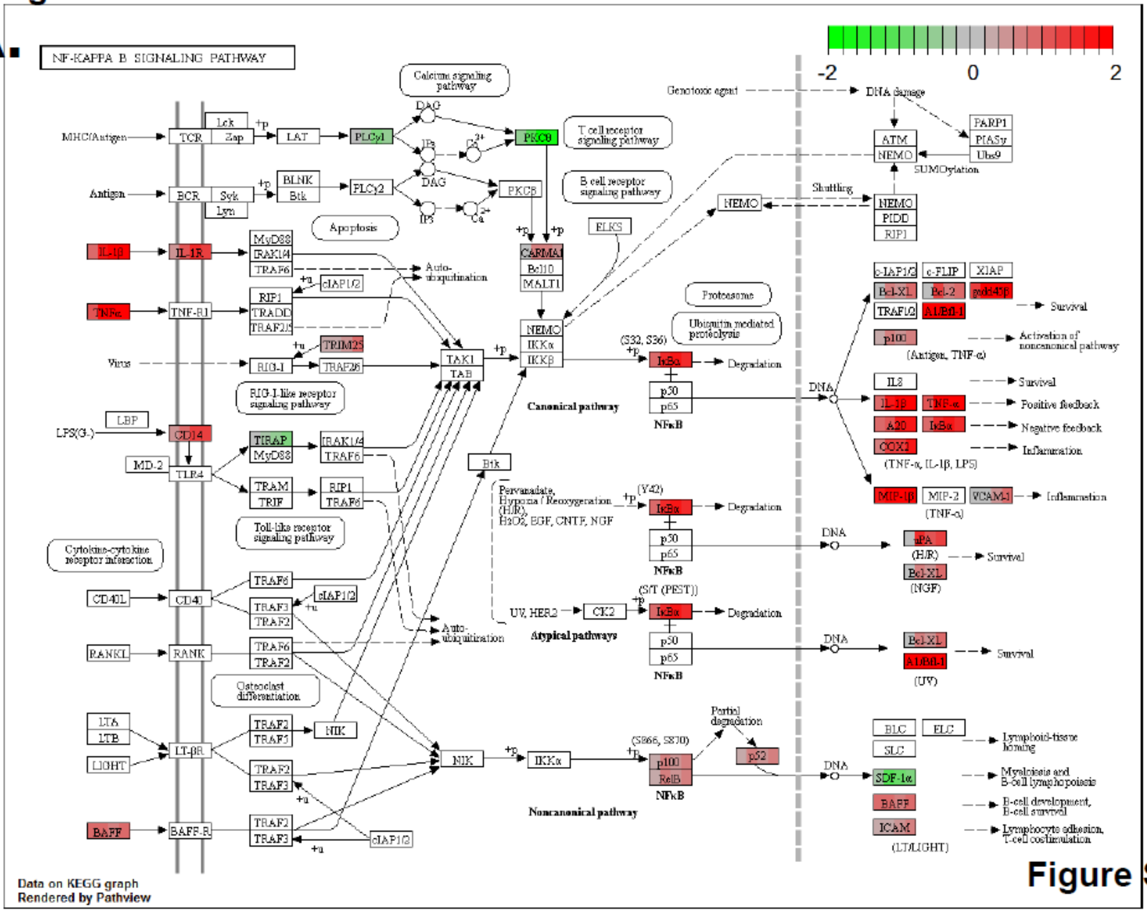


Figure S3

Wang H. et al

B.

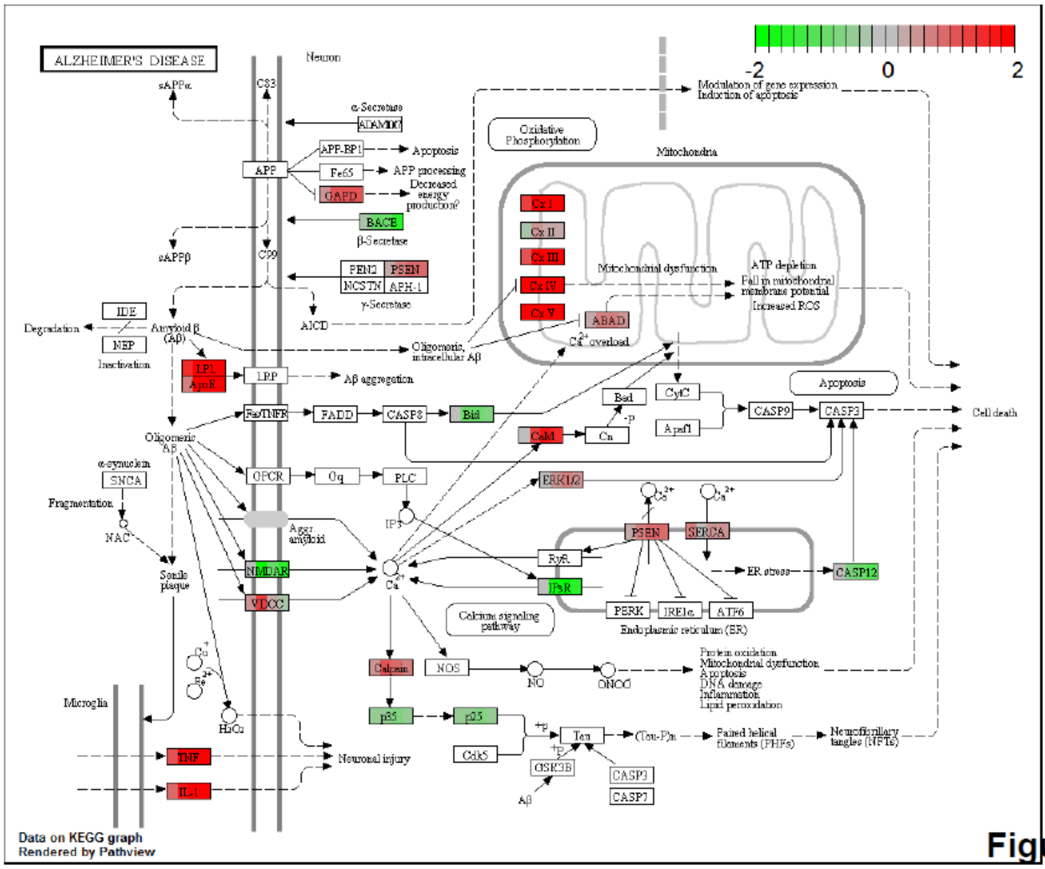


Figure S3
